# Waning anti-SARS-CoV-2 receptor-binding domain total antibody in CoronaVac-vaccinated individuals in Indonesia

**DOI:** 10.12688/f1000research.109676.2

**Published:** 2023-04-12

**Authors:** Harapan Harapan, Hibban Ar Royan, Islam Ing Tyas, Auda Nadira, Irham Faraby Abdi, Samsul Anwar, Milda Husnah, Ichsan Ichsan, Agung Pranata, Mudatsir Mudatsir, Maimun Syukri, Samsul Rizal, Razali ., Hamdani ., Rudi Kurniawan, Irwansyah Irwansyah, Sarwo Edhy Sofyan

**Affiliations:** 1 Medical Research Unit, School of Medicine, Universitas Syiah Kuala, Banda Aceh, 23111, Indonesia; 2 Department of Microbiology, School of Medicine, Universitas Syiah Kuala, Banda Aceh, 23111, Indonesia; 3 Tropical Disease Centre, School of Medicine, Universitas Syiah Kuala, Banda Aceh, 23111, Indonesia; 4 Department of Statistics, Faculty of Mathematics and Natural Sciences, Universitas Syiah Kuala, Banda Aceh, 23111, Indonesia; 5 Tsunami and Disaster Mitigation Research Center (TDMRC), Universitas Syiah Kuala, Banda Aceh, 23111, Indonesia; 6 Department of Parasitology, School of Medicine, Universitas Syiah Kuala, Banda Aceh, 23111, Indonesia; 7 Department of Internal Medicine, School of Medicine, Universitas Syiah Kuala, Banda Aceh, 23111, Indonesia; 8 Department of Mechanical and Industrial Engineering, Faculty of Engineering, Universitas Syiah Kuala, Banda Aceh, 23111, Indonesia

**Keywords:** COVID-19, neutralizing antibody, CoronaVac, anti-SRBD, Sinovac

## Abstract

**Background**: The decrease of immunity acquired from COVID-19 vaccines is a potential cause of breakthrough infection. Understanding the dynamics of immune responses of vaccine-induced antibodies post-vaccination is important. This study aimed to measure the level of anti-SARS-CoV-2 receptor-binding domain (RBD) total antibody in individuals at different time points upon the receipt of the second dose of CoronaVac vaccine, as well as evaluate the plausible associated factors.

**Methods**: A cross-sectional study was conducted among CoronaVac-vaccinated residents in Banda Aceh, Indonesia. The level of anti-SARS-CoV-2 RBD total antibody was measured using Elecsys immunoassay. A set of standardized and validated questionnaires were used to assess the demographics and other associated factors.

**Results**: Our results showed waning anti-SARS-CoV-2 RBD total antibody titres over time post-vaccination. Compared to samples of the first month post-vaccination, the antibody titres were significantly lower than those of five-months (mean 184.6 vs. 101.8 U/mL, p = 0.009) and six-months post-vaccination (mean 184.6 vs. 95.59 U/mL, p = 0.001). This suggests that the length of time post-vaccination was negatively correlated with titre of antibody. A protective level of antibody titres (threshold of 15 U/mL) was observed from all the samples vaccinated within one to three months; however, only 73.7% and 78.9% of the sera from five- and six-months possessed the protective titres, respectively. The titre of antibody was found significantly higher in sera of individuals having a regular healthy meal intake compared to those who did not (mean 136.7 vs. 110.4 U/mL, p = 0.044), including in subgroup analysis that included those five to six months post-vaccination only (mean 79.0 vs. 134.5 U/mL, p = 0.009).

**Conclusions**: This study provides insights on the efficacy of CoronaVac vaccine in protecting individuals against SARS-CoV-2 infection over time, which may contribute to future vaccination policy management to improve and prolong protective strategy.

## Introduction

Prevention of severe coronavirus disease 2019 (COVID-19), caused by severe acute respiratory syndrome coronavirus 2 (SARS-CoV-2) and its associated mortality is still a major hurdle worldwide. The disease is associated with several long-term consequences. ^1^^,^^2^ To date, no truly effective antivirals or therapeutic strategy has been successfully developed for the treatment of critical COVID-19. Some drugs, such as remdesivir and hydroxychloroquine, showed limited efficacy. ^3^^,^^4^ The presence of individual protective immunity, instead, could provide effective protection against acute SARS-CoV-2 infection. ^5^ Therefore, understanding and profiling antibody response towards SARS-CoV-2 is highly prominent as it will provide significant insight into therapeutic approaches. Specific antibodies detection, as an indirect method for COVID-19 diagnosis, allows the evaluation of seroprevalence, promoting a better understanding of the COVID-19 transmission among communities. It also enables the identification of individuals potentially invulnerable to SARS-CoV-2 infection, monitoring of herd immunity, as well as formulating strategies for global COVID-19 vaccination. ^6^^–^^8^

Neutralizing antibodies (NAbs) provide real protective immunity as they play a crucial role in hampering the binding of the SARS-CoV-2 receptor-binding domain (RBD) of surface spike (S) protein to the human angiotensin-converting enzyme 2 (ACE2) receptor, ^9^^–^^12^ blocking viral infection and minimizing disease severity. Therefore, measuring SARS-CoV-2 NAbs is considered an acceptable approach for the analysis of protective immune response against COVID-19 after vaccination. ^13^

Vaccination triggers the neutralizing immune response, making vaccination an effective strategy to control virus-associated diseases although the acceptance rate varies among countries. ^14^^,^^15^ Vaccination programs for COVID-19 using various vaccine types have therefore been vigorously conducted, including the inactivated CoronaVac. ^16^ Preclinical studies revealed the ability of CoronaVac to induce NAb production as well as providing partial and complete protection in the tested animals against COVID-19. ^17^ This vaccine has also been reportedly well tolerated and induced the humoral immune responses in individuals aged 18–59 years. ^18^ However, given the fact that reinfection still occurred in individuals vaccinated with CoronaVac and the vaccine-induced NAb titres have been reportedly waning over time, it is questionable to what extent this vaccine can persist and protect against SARS-CoV-2. Therefore, evaluating NAb response in individuals within a different duration of time upon CoronaVac vaccination is critically important, as it may serve as a predictor of vaccine protection efficacy. This study sought to evaluate the titre of anti-SARS-CoV-2 RBD total antibody (IgG and IgM) among CoronaVac-vaccinated individuals as well as to determine the potential factors associated with the level of the titre.

## Methods

### Study design and setting

A cross-sectional study among COVID-19-vaccinated residents in Banda Aceh, Indonesia was conducted from May to July 2021. The subjects were post COVID-19-vaccinated individuals residing in Banda Aceh who met the inclusion criteria. Individuals vaccinated with two doses of CoronaVac vaccine (Sinovac Biotech) within one to six months prior to the recruitment period, had never been diagnosed with COVID-19, and aged between 18 and 65 years were considered eligible for the study. Previous SARS-CoV-2 infection was confirmed if the individual had positive RT-PCR or SARS-CoV-2 antigen test and recorded in Indonesian COVID-19 National Registry. Individuals who had COVID-19 after the vaccination or having symptoms during the period of recruitment and having malignant diseases were excluded. The recruitment was conducted based on COVID-19 vaccination records obtained from Prince Nayef Hospital Universitas, Syiah Kuala, Banda Aceh, Indonesia. The minimum sample size was calculated using ClinCalc sample size calculator. The minimum sample size was 112 individuals with assumption that the difference of the antibody anti-SARS-CoV-2 between two months is around 18 U/mL; with alpha 0.05 and study power of 80%.

### Study variables

The response variable was the level of anti-SARS-CoV-2 RBD total antibody after CoronaVac vaccination, measured using the electrochemiluminescence immunoassay method. Several plausible factors that might be associated with the titre of anti-SARS-CoV-2 RBD total antibody were measured and collected. This included demographic characteristics such as age and gender, body mass index (BMI), history of illness, history of immunization (BCG and influenza), adverse events following vaccination (allergy, fever, arthralgia, and acute paralysis), exercise routine, smoking status, comorbidities (hypertension, diabetes, hyperlipidaemia, chronic obstructive pulmonary disease (COPD), asthma, and gout), sleep quality, and level of stress. All those information were collected from each individual using direct face-to-face interview. BMI was measured by measuring participant height and weight. A set of standardized and validated questionnaires were used: (a) the quality of sleep was assessed according to Pittsburgh Sleep Quality Index (PSQI) ^19^; and (b) the level of stress was determined based on the Depression Anxiety Stress Scales 42 (DASS-42). ^20^

### Quantitation of anti-SARS-CoV-2 RBD total antibody

Approximately 3 mL of venous blood was collected and centrifuged to separate the sera. The sera were stored at -80°C until used. Anti-SARS-CoV-2 RBD total antibody level was measured using an electrochemiluminescence immunoassay method using Elecsys ^®^ Anti-SARS-CoV-2 S immunoassay following the manufacturer’s instruction (Roche Diagnostics International Ltd, Rotkreuz, Switzerland). The assay was conducted using an automatic Roche cobas ^®^ E411 immunoassay analyzer (Roche Diagnostics International Ltd, Rotkreuz, Switzerland). The assay uses a recombinant protein representing the RBD of the S antigen in a double-antigen sandwich assay approach. In brief, 20 μL of sample incubated with SARS-CoV-2-Ag~biotin (a biotinylated SARS-CoV-2 RBD-specific recombinant antigen) and SARS-CoV-2 Ag~Ru (bpy) (SARS-CoV-2 RBD-specific recombinant antigen labeled with a ruthenium complex) instruction (Roche Diagnostics International Ltd, Rotkreuz, Switzerland). The complex became bound to the solid phase after streptavidin-coated microparticles were added. The reaction mixture was aspirated into the measuring cell where the microparticles are magnetically captured onto the surface of the electrode. The chemiluminescent emission was then measured by a photomultiplier. The titres of the anti-SARS-CoV-2 RBD total antibody were then classified as protective and non-protective using a cut-off 15 U/mL. ^21^

### Statistical analysis

Analysis of variance (Anova) and Student t-test were used to compare the titres of anti-SARS-CoV-2 RBD total antibody between demographic groups as appropriate. Linear regression was employed to determine factors affecting the anti-SARS-CoV-2 RBD total antibody titre. Pearson correlation was used to assess the correlation between day of post vaccination, age and BMI with the titres of anti-SARS-CoV-2 RBD total antibody. The analyses were conducted using SPSS version 20 (IBM SPSS Inc., Chicago, IL, USA) (RRID:SCR_019096).

### Ethical approval

This study was approved by the Health Research Ethics Committee of the Faculty of Medicine, Universitas Syiah Kuala - Zainoel Abidin Hospital (#198/EA/FK-RSUDZA/2021 and KEPPKN Registration #1171012P). All the participants were informed of the study procedures and provided written consent prior to participating in this study.

## Results

### The level of anti-SARS-CoV-2 RBD total antibody

**Figure 1.**
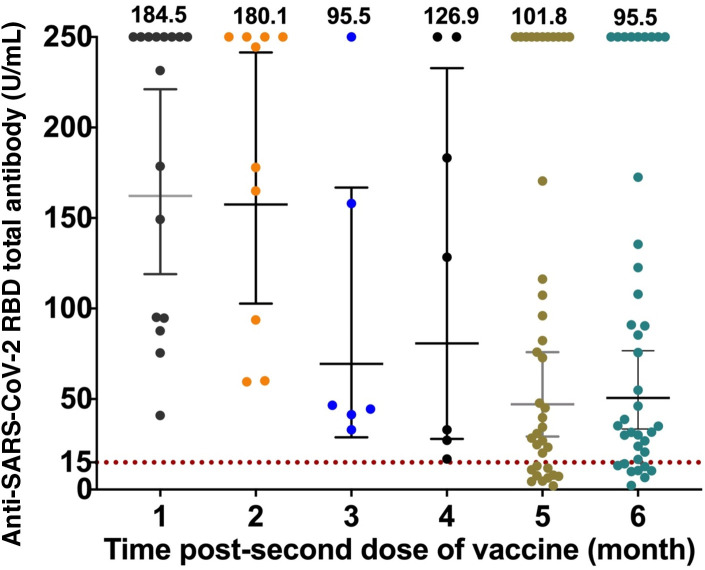
The individual titres and the mean titres of anti-SARS-CoV-2 RBD total antibody of different times of post vaccination.

We measured the titre of anti-SARS-CoV-2 RBD total antibody in individuals with different length of time post the second dose. The individual titres and the mean titres of anti-SARS-CoV-2 RBD total antibody from each group are presented in Figure 1. The mean of titre of the one-month group was 184.5 U/mL and the mean decreased to 101.8 U/mL in samples collected from those five-month post-vaccination and to 95.5 U/mL in the six-month group. All samples from one to three months post-vaccination had protective anti-SARS-CoV-2 RBD total antibody titres (more than 15 U/mL). However, only 78.9% of the samples from six-months post-vaccination had a protective level of anti-SARS-CoV-2 RBD total antibody titres.

### Factor associated with the level of anti-SARS-CoV-2 RBD total antibody

We assessed the association of some plausible factors with the titre of anti-SARS-CoV-2 RBD total antibody ( Table 1). Our data suggested that healthy meal intake was associated with the titres of anti-SARS-CoV-2 RBD total antibody. The titre of anti-SARS-CoV-2 RBD total antibody was higher in those who took regular healthy meal compared to those who did not (136.7 vs. 110.4 U/mL, p = 0.044) ( Table 1). When we included only those who were vaccinated within five to six months (n = 76), only regular healthy meal intake was associated with the level of anti-SARS-CoV-2 RBD total antibody (79.0 vs 134.5 U/mL) ( Table 2).

**Table 1.** Linear regression showing the predictor of the level of anti-SARS-CoV-2 RBD total antibody post-vaccination (n = 115).

Characteristic	n (%)	Mean concentration (±SD), U/mL	Initial model		Final model	
			ß (95% CI)	*p*–value	ß (95% CI)	*p*–value
Post-vaccination time (month)						
1 (Reference, *R*)	16 (13.9)	184.6 (±79.9)				
2	10 (8.7)	180.1 (±81.9)	12.9 (-75.1, 100.9)	0.771	9.8 (-65.7, 85.3)	0.797
3	6 (5.2)	95.6 (±89.0)	-57.5 (-159.1, 44.0)	0.263	-99.8 (-188.6, -11.1)	0.028
4	7 (6.1)	127.0 (±103.5)	-49.9 (-146.1, 46.3)	0.305	-50.6 (-134.1, 32.9)	0.232
5	38 (33.0)	101.8 (±102.2)	-70.3 (-134.9, -5.7)	0.033	-74.3 (-129.5, -19.1)	0.009
6	38 (33.0)	95.6 (±95.2)	-96.2 (-155.8, -36.7)	0.002	-93.3 (-148.2, -38.4)	0.001
Age (year)						
20-30 (R)	59 (51.3)	122.0 (±93.7)				
31-40	32 (27.8)	127.3 (±109.5)	3.5 (-49.4, 56.3)	0.896		
41-50	16 (13.9)	117.5 (±108.4)	-4.9 (-70.4, 60.7)	0.883		
>50	8 (7.0)	71.0 (±80.6)	-6.5(-100.3, 87.4)	0.892		
Gender						
Male (R)	38 (33.0)	121.9 (±102.2)				
Female	77 (67.0)	118.0 (±98.4)	-34.3 (-83.8, 15.2)	0.172		
Body mass index (BMI)						
Underweight (R)	11 (9.6)	102.0 (±99.1)				
Normal	35 (30.4)	125.8 (±96.4)	13.9 (-57.1, 85.0)	0.698		
Overweight	26 (22.6)	103.2 (±99.2)	12.3 (-64.2, 88.9)	0.750		
Obesity	43 (37.4)	128.2 (±103.4)	24.5 (-47.3, 96.3)	0.500		
Regular exercise						
Yes	32 (27.8)	103.2 (±98.3)	2.2 (-47.7, 52.1)	0.930		
No (R)	83 (72.2)	125.5 (±99.5)				
Sleep quality						
Good	40 (34.8)	98.1 (±95.1)	-45.5 (-87.6, -3.4)	0.035	-30.0 (-66.6, 6.6)	0.107
Poor (R)	75 (65.2)	130.6 (±100.2)				
Healthy meal						
No	76 (66.1)	110.4 (±98.8)	-46.7 (-93.4, 0.1)	0.050	-39.2 (-77.3, -1.0)	0.044
Yes (R)	39 (33.9)	136.7 (±99.2)				
Regular food supplementation						
Yes	65 (56.5)	116.6 (±98.5)	15.3 (-25.2, 55.8)	0.454		
No (R)	50 (43.5)	122.8(±101.1)				
Smoking						
Yes (R)	10 (8.7)	100.1 (±107.1)				
No	105 (91.3)	121.1 (±98.9)	20.4 (-63.4, 104.2)	0.630		
Stress level						
Normal (R)	96 (83.5)	123.2 (±101.5)				
Mild	5 (4.3)	76.1 (±97.8)	-81.6 (-182.2, 18.9)	0.110		
Moderate	7 (6.1)	88.6 (±74.0)	-66.8 (-153.7, 20.2)	0.131		
Severe	7 (6.1)	126.8 (±96.4)	1.75 (-77.0, 80.5)	0.965		
Stress						
Yes	19 (16.5)	99.4 (±86.8)	NA (NA, NA)	NA		
No (R)	96 (83.5)	123.2 (±101.5)				
Comorbidity						
Hypertension						
Yes (R)	6 (5.2)	45.1 (±46.3)				
No	109 (94.8)	123.4 (±99.9)	65.5 (-25.3, 156.3)	0.155		
Hyperlipidaemia						
Yes (R)	8 (7.0)	53.0 (±80.4)				
No	107 (93.0)	124.2 (±99.1)	44.6 (-42.1, 131.3)	0.309		
Gout						
Yes (R)	13 (11.3)	103.9 (±94.1)				
No	102 (88.7)	121.2 (±100.2)	35.1 (-39.6, 109.7)	0.353		
History of infection except COVID-19						
Yes (R)	15 (13.0)	135.9 (±94.7)	18.4 (-43.5, 80.3)	0.556		
No	100 (87.0)	116.8 (±100.2)				
Experience of vaccination side effect						
No (R)	58 (50.4)	118.2 (±98.8)				
Yes	57 (49.6)	120.4 (±100.7)	4.9 (-36.7, 46.5)	0.815		
History of flu vaccination						
Yes	24 (20.9)	107.1 (±96.0)	-13.9 (-63.2, 35.4)	0.577		
No (R)	91 (79.1)	122.5 (±100.4)				

**Table 2.** Linear regression showing the predictor of the level of anti-SARS-CoV-2 RBD total antibody after 5-6 months post-vaccination (n = 76).

Characteristic	n (%)	Mean concentration (±SD), U/ml	Initial model		Final model	
			ß (95% CI)	*p*–value	ß (95% CI)	*p*–value
Post-vaccination time (month)						
5 (R)	38 (50.0)	101.8 (±102.2)				
6	38 (50.0)	95.6 (±95.2)	-30.2 (-86.8, 26.3)	0.289		
Age (year)						
20-30 (R)	34 (44.7)	103.6 (±93.6)				
31-40	26 (34.2)	114.4 (±111.8)	-6.6 (-71.1, 57.9)	0.838		
41-50	11 (14.5)	72.0 (±91.7)	-69.9 (-158.3, 18.5)	0.119		
>50	5 (6.6)	42.7 (±39.3)	-14.9 (-178.1, 148.2)	0.855		
Gender						
Male (R)	25 (32.9)	100.9 (±102.3)				
Female	51 (67.1)	97.6 (±97.1)	-3.0 (-68.6, 62.6)	0.928		
Body mass index (BMI)						
Underweight (R)	8 (10.5)	85.4 (±102.8)				
Normal	18 (23.7)	86.2 (±87.1)	24.2 (-65.0, 113.4)	0.588		
Overweight	21 (27.6)	92.7 (±97.8)	47.0 (-49.8, 143.7)	0.334		
Obesity	29 (38.2)	114.5 (±106.3)	33.6 (-56.6, 123.7)	0.459		
Regular exercise						
Yes	24 (31.6)	92.4 (±98.4)	19.2 (-45.4, 83.8)	0.553		
No (R)	52 (68.4)	101.6 (±98.9)				
Sleep quality						
Good	27 (35.5)	82.4 (±95.6)	-40.0 (-96.2, 16.1)	0.159		
Poor (R)	49 (64.5)	107.7 (±99.4)				
Healthy meal						
No	49 (64.5)	79.0 (±89.6)	-78.1 (-142.6, -13.7)	0.018	-62.3 (-108.5, -16.1)	0.009
Yes (R)	27 (35.5)	134.5 (±104.5)				
Regular food supplementation						
Yes	48 (63.2)	94.4 (±95.4)	23.0 (-32.2, 78.2)	0.407		
No (R)	28 (36.8)	106.1 (±104.1)				
Smoking						
Yes (R)	6 (7.9)	91.7 (±123.2)				
No	70 (92.1)	99.3 (±96.8)	-14.8 (-122.8, 93.3)	0.785		
Stress level						
Normal (R)	66 (86.8)	104.2 (±103.0)				
Mild	3 (3.9)	34.4 (±13.8)	-162.5 (-296.7, -28.2)	0.019	-90.6 (-203.3, 22.2)	0.114
Moderate	3 (3.9)	76.9 (±19.2)	-37.3 (-166.9, 92.2)	0.566	-48.0 (-160.7, 64.7)	0.399
Severe	4 (5.3)	73.2 (±65.5)	-27.5 (-139.9, 84.9)	0.626	-25.8 (-123.3, 71.7)	0.599
Stress						
Yes	10 (132)	62.7 (±44.0)	NA (NA, NA)	NA		
No (R)	66 (86.8)	104.2 (±103.0)				
Comorbidity						
Hypertension						
Yes (R)	5 (6.6)	45.2 (±51.7)				
No	71 (93.4)	102.5 (±99.8)	66.9 (-49.8, 183.6)	0.255		
Hyperlipidaemia						
Yes (R)	6 (7.9)	23.1 (±24.9)				
No	70 (92.1)	105.2 (±99.4)	55.5 (-78.2, 189.1)	0.409		
Gout						
Yes (R)	6 (7.9)	73.9 (±94.7)				
No	70 (92.1)	100.8 (±98.8)	11.4 (-109.3, 132.1)	0.851		
History of infection except COVID-19						
Yes (R)	7 (9.2)	91.7 (±85.1)	-3.2 (-93.0, 86.6)	0.943		
No	69 (90.8)	99.4 (±99.9)				
Experience of vaccination side effect						
No (R)	39 (51.3)	94.3 (±94.1)				
Yes	37 (48.7)	103.4 (±103.4)	4.9 (-53.4, 63.2)	0.867		
History of flu vaccination						
Yes	15 (19.7)	65.5 (±69.5)	-53.0 (-117.9, 11.9)	0.108		
No (R)	61 (80.3)	106.9 (±102.8)				

Our data suggest that the length of post-vaccination time was associated with the titres of anti-SARS-CoV-2 RBD total antibody after adjustment ( Table 1). Compared to those in first month of vaccination, the level of anti-RBD total antibody were significantly lower from individuals five months-post vaccination (184.6 *vs.* 101.8 U/mL, p = 0.009). Similarly, the level of anti-RBD total antibody titres was significantly lower in samples from individuals six-months post vaccination (184.6 *vs.* 95.59 U/mL, p = 0.001) ( Table 1). Spearman’s rank correlation ( *r _s_*) also showed that the time of post vaccination was correlated negatively with titre of anti-RBD total antibody ( Table 3). This indicated that the longer time post-vaccination the lower total antibody anti-SRBD titre.

**Table 3.** Spearman’s rank correlation ( *r _s_*) showing predictor of anti-SARS-CoV-2 RBD total antibody titre post-vaccination (n = 115).

Correlation of variables	r _s_ (95%CI)	*p*-value
Time of post vaccination (day) - total antibody anti-RBD titre	-0.273 (-0.437, -0.091)	0.003
Age - total antibody anti-RBD titre	-0.135 (-0.311, 0.050)	0.150
BMI - total antibody anti-RBD titre	-0.030 (-0.212, 0.154)	0.750

## Discussion

Our results revealed waning anti-SARS-CoV-2 RBD total antibody in individuals after vaccination with CoronaVac vaccine. We noted a significant decline in the level of anti-SARS-CoV-2 RBD total antibody five and six months after the receipt of the second dose of the vaccine when compared to the first month ( Figure 1 and Table 1), suggesting that the time of post vaccination was negatively correlated with total antibody anti-SRBD titre. Reduction in vaccine-induced neutralization titres within the first six months upon the second dose vaccination has also been previously reported in several different vaccines. ^22^^–^^25^ However, a similar study suggested that the decrease in CoronaVac-induced anti-S antibodies levels was faster in individuals without prior SARS-CoV-2 infection compared to those with previous infection. ^26^ NAbs can persist in the body for two to 12 months after the infection onset. ^5^^,^^27^ This might suggest that CoronaVac would assumingly provide greater and longer-lasting protective impact when administered in previously infected individuals, as it may boost the memory immune cells that developed following the infection. ^27^^,^^28^

The underlying causes of rapid waning of vaccine induced anti-SARS-CoV-2 RBD total antibody remains to be determined. However, several studies reported that waning titres have been associated with IgG anti-RBD immune response ^29^^,^^30^ and neutralizing capacity was positively correlated with IgG antibody titres. ^31^ Furthermore, the loss of short-lived plasma cells has been considered the cause of initial rapid waning of antibodies in SARS-CoV-2 infected individuals in general, while establishment of long-lived plasma was thought to contribute to the elevation of antibody level. ^32^^,^^33^ Defective Bcl6+ follicular T-cells due to the absence of germinal centers in the thoracic lymph nodes in dead COVID-19 patients was proposedly unable to activate memory B-cells, leading to a decrease in long-lasting and high-affinity antibody production. ^34^ This mechanism has been suggested as a potential explanation regarding rapid antibody decline in SARS-CoV-2. ^34^

This study suggested that more than 20% of the sample of five- and six-month post-vaccination had anti-SARS-CoV-2 RBD total antibody titres <15 U/mL compared to those of one to three months, suggesting a possible loss of protection after three months of vaccination against SARS-CoV-2 ( Table 2). Reduction in the level of protective antibody might be due to the decreased titres as waning antibody titres have been reportedly correlated with reduced protection over time. ^12^^,^^25^^,^^35^ This remarkable reduction in the titre of anti-SARS-CoV-2 RBD total antibody and its decline in protective level might indicate the need for an additional booster dose of CoronaVac vaccine to protect against COVID-19 among individuals without prior SARS-CoV-2 infection.

Our findings suggested the level of anti-SARS-CoV-2 RBD total antibody was significantly associated with a regular intake of healthy meals, regardless of the duration post-vaccination ( Tables 1 and 2). An adequate intake of vitamins such as vitamin A, B12, B6, and C, zinc, as well as iron is suggested to maintain immune function, particularly during COVID-19. ^36^ Vitamin C has been known to boost the immune system and prevent any viral infection. ^36^^,^^37^ Healthy meals and optimal nutritional intake will impact the immune system through cell activation, signalling molecule modification, and gene expression. A variety of dietary components also determines the composition of gut microbes which then form the immune response in the body. ^38^

They are some limitations of our study that should be discussed. We determined the titre of anti-SARS-CoV-2 RBD total antibody which might not exactly represent the NAb that should be assessed using the plaque reduction neutralization test (PRNT). However, anti-SARS-CoV-2 RBD total antibody has good agreement with PRNT test. ^39^ In this present study, we classified the individual had previous SARS-CoV-2 infection if they had positive RT-PCR or SARS-CoV-2 antigen test. Therefore, there is possibility that SARS-CoV-2-infected individuals still be included as samples if they have not tested. In this study, we only determined anti-SARS-CoV-2 RBD total antibody among those who completed the primary doses of CoronaVac; therefore further study measuring the anti-RBD antibody after the booster dose of CoronaVac or other types of COVID-19 vaccines will provide better understanding of the antibody dynamics.

## Conclusions

Our data indicated that the level of anti-SARS-CoV-2 RBD total antibody dropped significantly within five and six months after the second dose of CoronaVac vaccination, along with the decay of protective capacity in several samples. Our study suggested that the length of time post-vaccination negatively correlated with the titre of anti-SARS-CoV-2 RBD total antibody. Regular healthy meal intake was associated significantly with the level of anti-RBD total antibody, regardless of the duration post-vaccination. This provided a prediction of CoronaVac vaccine efficacy in protecting individuals against SARS-CoV-2 infection over time upon the second dose vaccination. This may contribute to future vaccination policy management to improve and prolong the protective strategy through vaccination.

## Data availability

### Underlying data

Figshare: Underlying data for ‘Waning anti-SARS-CoV-2 neutralizing antibody in CoronaVac-vaccinated individuals in Indonesia’. https://doi.org/10.6084/m9.figshare.19149797. ^40^

This project contains the following underlying data:

- Data file: Master Data.xlsx [Table containing the raw data of the study]

### Reporting guidelines

Figshare: STROBE checklist for ‘Waning anti-SARS-CoV-2 neutralizing antibody in CoronaVac-vaccinated individuals in Indonesia'. https://doi.org/10.6084/m9.figshare.19149806. ^41^

Data are available under the terms of the Creative Commons Attribution 4.0 International license (CC-BY 4.0).

## Authors’ contributions

Harapan Harapan was responsible for the entire study setting, procedures, data presentation and preparing the manuscript. Hibban Ar Royan, Islam Ing Tyas, Auda Nadira and Irham Faraby Abdi were responsible in conducting the study, recruited the samples, collected the data and collected the blood samples as well as validating the results. Samsul Anwar did the analysis and was responsible in data analysis and validating the results. Milda Husnah contributed in project administration, manuscript preparation and validation of results. Ichsan Ichsan and Agung Pranata contributed in providing some resources during the study including lab facilities and responsible in validating the results. Mudatsir Mudatsir and Maimun Syukri provided the resources during the study including lab facilities and consolidated during external laboratory works and supervised the study. Samsul Rizal was the PI of the umbrella project of which this present study belonged to, contributed in study design of the project, supervised the study and validated the findings by participating in regular lab meetings, was responsible in budgeting of the project and as person in charge with the funding body. Razali, Hamdani, Irwansyah Irwansyah and Sarwo Edhy Sofyan were co-PIs of the umbrella project of which this present study belonged to, contributed in study design of the project and supervised the study and validated the findings by participating in regular lab meetings. Rudi Kurniawan was co-PIs of the umbrella project of which this present study belonged to, contributed in study design of the project, supervised the study and validated the findings by participating in regular lab meetings and was the project manager of the umbrella project. All authors have read the final manuscript and agreed for its submission to the journal.
