# Supplementary material for: Waning anti-SARS-CoV-2 receptor-binding domain total antibody in CoronaVac-vaccinated individuals in Indonesia
Source: F1000Res. 2023 Apr 12;11:300. Originally published 2022 Mar 10. [Version 2] doi: 10.12688/f1000research.109676.2 (PMC10209622; doi:10.12688/f1000research.109676.2)
Supplement: Peer review history

# Peer review: Reviewer response for version 2

**DOI:** 10.5256/f1000research.146484.r169538

**Published:** 2023-05-24

**Authors:** Md. Rabiul Islam

**Affiliations:** 1 Department of Pharmacy, University of Asia Pacific, Dhaka, Bangladesh

The manuscript has significantly improved after this revision. I think it now okay.

Is the work clearly and accurately presented and does it cite the current literature?

Yes

If applicable, is the statistical analysis and its interpretation appropriate?

Yes

Are all the source data underlying the results available to ensure full reproducibility?

Yes

Is the study design appropriate and is the work technically sound?

Yes

Are the conclusions drawn adequately supported by the results?

Yes

Are sufficient details of methods and analysis provided to allow replication by others?

Yes

Reviewer Expertise:

NA

I confirm that I have read this submission and believe that I have an appropriate level of expertise to confirm that it is of an acceptable scientific standard.

# Peer review: Reviewer response for version 2

**DOI:** 10.5256/f1000research.146484.r169537

**Published:** 2023-04-24

**Authors:** Ritthideach Yorsaeng

**Affiliations:** 1 Center of Excellence in Clinical Virology, Department of Pediatrics, Faculty of Medicine, Chulalongkorn University, Bangkok, Thailand

After reading a revised version. The quality was improved and flaw points were corrected.

 I found a minor error in the statistical analysis.

1. Analysis of variance (Anova). Suggest using "ANOVA" instead of "Anova" because this word is an abbreviation.

Is the work clearly and accurately presented and does it cite the current literature?

Partly

If applicable, is the statistical analysis and its interpretation appropriate?

Yes

Are all the source data underlying the results available to ensure full reproducibility?

Yes

Is the study design appropriate and is the work technically sound?

Partly

Are the conclusions drawn adequately supported by the results?

Yes

Are sufficient details of methods and analysis provided to allow replication by others?

Yes

Reviewer Expertise:

SARS-CoV-2 immunology, SARS-CoV-2 vaccine trial.

I confirm that I have read this submission and believe that I have an appropriate level of expertise to confirm that it is of an acceptable scientific standard.

# Peer review: Reviewer response for version 1

**DOI:** 10.5256/f1000research.121210.r138940

**Published:** 2022-05-30

**Authors:** Ritthideach Yorsaeng

**Affiliations:** 1 Center of Excellence in Clinical Virology, Department of Pediatrics, Faculty of Medicine, Chulalongkorn University, Bangkok, Thailand

1. The immunological assessment used in this manuscript is not "truly" neutralising antibody assay. Because the Elecsys Anti-SRAS-CoV-2 S is the "binding" assay in contrast to neutralizing assay is the "blocking" assay. The reagent of the binding assay is binding to the RBD regardless of the functional, conformational and location of the trimeric spike-RBD. That's why the test is not very strongly correlated to the neutralising assay.
  The result is implied to NAb level, not representative of NAb level. Suggesting to use another word than NAb.
  Please see also:
  - https://www.nature.com/articles/s41598-022-07849-2/figures/4
  - https://www.thelancet.com/journals/ebiom/article/PIIS2352-3964(20)30477-1/fulltext
2. What is the IU/mL? The Elecsys Anti-SARS-CoV-2 S has a unit as U/mL or converted to BAU/mL by the conversion factor of 1.028. 
3. The Study variables section was mentioned "ELISA", but the result was not show in the result.

Is the work clearly and accurately presented and does it cite the current literature?

Partly

If applicable, is the statistical analysis and its interpretation appropriate?

Yes

Are all the source data underlying the results available to ensure full reproducibility?

Yes

Is the study design appropriate and is the work technically sound?

Partly

Are the conclusions drawn adequately supported by the results?

Yes

Are sufficient details of methods and analysis provided to allow replication by others?

Yes

Reviewer Expertise:

SARS-CoV-2 immunology, SARS-CoV-2 vaccine trial.

I confirm that I have read this submission and believe that I have an appropriate level of expertise to state that I do not consider it to be of an acceptable scientific standard, for reasons outlined above.

## References

[1] : Neutralizing antibodies against SARS-CoV-2 variants of concern elicited by the comirnaty COVID-19 vaccine in nursing home residents. *Scientific Reports* .2022;12(1) : 10.1038/s41598-022-07849-2 10.1038/s41598-022-07849-2 35260713PMC8904770

[2] : Analytical and clinical performances of five immunoassays for the detection of SARS-CoV-2 antibodies in comparison with neutralization activity. *EBioMedicine* .2020;62: 10.1016/j.ebiom.2020.103101 103101 10.1016/j.ebiom.2020.103101 33160207PMC7640894

# Response

**Published:** 2023-04-01

**Authors:** Harapan Harapan

**Affiliations:** Syiah Kuala University, Indonesia

Dear Reveiwer,

 Thank you for your valuable comments. We have revised our article based on your comments. Detailed responses are presented below: 

 1. The immunological assessment used in this manuscript is not "truly" neutralising antibody assay. Because the Elecsys Anti-SRAS-CoV-2 S is the "binding" assay in contrast to neutralizing assay is the "blocking" assay. The reagent of the binding assay is binding to the RBD regardless of the functional, conformational and location of the trimeric spike-RBD. That's why the test is not very strongly correlated to the neutralising assay.

 The result is implied to NAb level, not representative of NAb level. Suggesting to use another word than NAb.

 Please see also:

- https://www.nature.com/articles/s41598-022-07849-2/figures/4
- https://www.thelancet.com/journals/ebiom/article/PIIS2352-3964(20)30477-1/fulltex

**RESPONSE: **We have changed the term in the entire article from NAb to "anti-SARS-CoV-2 RBD total antibody" **.** We would like to thank you for this constructive comment. 

 2. What is the IU/mL? The Elecsys Anti-SARS-CoV-2 S has a unit as U/mL or converted to BAU/mL by the conversion factor of 1.028. 

**RESPONSE**: Thank you for picking up this typo. It should be U/mL not IU/mL. We have corrected the typos related to this.

 ​​​

 3. The Study variables section was mentioned "ELISA", but the result was not show in the result.

**RESPONSE**:  The ELISA in this section should ECLIA and has been corrected to: "The response variable was the level of anti-SARS-CoV-2 RBD total antibody after CoronaVac vaccination, measured using the electrochemiluminescence immunoassay method." 

 Best regards

 Authors

# Peer review: Reviewer response for version 1

**DOI:** 10.5256/f1000research.121210.r128986

**Published:** 2022-05-20

**Authors:** Md. Rabiul Islam

**Affiliations:** 1 Department of Pharmacy, University of Asia Pacific, Dhaka, Bangladesh

Thank you for this opportunity to review this timely work in the field of immunology. Here the authors described how vaccine induced immunity would decrease and that might cause further massive infection due to Coronavirus. They show the level of neutralizing antibody (NAb) among vaccinated individuals at different points of time after getting vaccines. This is an interesting and valuable study to predict future effects of coronavirus vaccines to prevent potential infections. However, I have some observations that I believe would help to improve the manuscript. My specific points are below-

 Major points

1. How do the authors exclude the levels of infection induced neutralizing antibody (NAb) among the participants? Was there any way to identify those people who had previous covid-19 infection but not diagnosed? This information will help the readers to know how much immunity acquired by vaccines and how this will diminish over time.
2. SARS-Cov-2 antibodies will automatically decrease over time and this is also true for most vaccines in other diseases, therefore, we take booster or additional doses. How the authors will justify this statement.
3. The practice of booster or additional dose is common for covid-19 and it would be better if the authors could see the levels of antibody decrease after the third/booster dose. If it is not possible this can be mentioned as a potential limitation of the present study.
4. What are the findings about other vaccines for Covid-19? Are there any studies that have assessed antibody levels among vaccinated peoples who have been given other vaccines?
5. How can we prevent the significant loss of vaccine acquired immunity? What the authors are proposing to prevent/prolong the immunity among vaccinated individuals?

 Minor points 

1. Please add a separate section just after discussion about the potential limitations and future direction based on the present findings considering the current weakness.
2. In conclusion, we should not repeat the results, there should have some implications of the present findings in theory and practice.

Is the work clearly and accurately presented and does it cite the current literature?

Yes

If applicable, is the statistical analysis and its interpretation appropriate?

Yes

Are all the source data underlying the results available to ensure full reproducibility?

Yes

Is the study design appropriate and is the work technically sound?

Yes

Are the conclusions drawn adequately supported by the results?

Yes

Are sufficient details of methods and analysis provided to allow replication by others?

Yes

Reviewer Expertise:

Public health, infection control and prevention, psychiatric epidemiology.

I confirm that I have read this submission and believe that I have an appropriate level of expertise to confirm that it is of an acceptable scientific standard, however I have significant reservations, as outlined above.

# Response

**Published:** 2023-04-01

**Authors:** Harapan Harapan

**Affiliations:** Syiah Kuala University, Indonesia

Dear Reveiwer,

 We would like to thank you for your valuable comments and suggestions for our article. We have revised our manuscript based on the comments and suggestions. Our responses for each comments and suggestions are follow: 

 Major points

 1. How do the authors exclude the levels of infection induced neutralizing antibody (NAb) among the participants? Was there any way to identify those people who had previous covid-19 infection but not diagnosed? This information will help the readers to know how much immunity acquired by vaccines and how this will diminish over time.

**RESPONSE:** We have added more information on this in Study design and setting: "Previous SARS-CoV-2 infection was confirmed if the individual had positive RT-PCR or SARS-CoV-2 antigen test and recorded in Indonesian COVID-19 National Registry." We have included this as one of our limitations. We stated: "In this present study, we classified the individual had previous SARS-CoV-2 infection if they had positive RT-PCR or SARS-CoV-2 antigen test. Therefore, there is possibility that SARS-CoV-2-infected individuals still be included as samples if they have not tested."

 2. SARS-CoV-2 antibodies will automatically decrease over time and this is also true for most vaccines in other diseases, therefore, we take booster or additional doses. How the authors will justify this statement.

**RESPONSE:** The speed of the depletion of the anti-SARS-CoV-2 is something that not well understood as well as whether the level is still protective of not and therefore this study was conducted. The depletion of the antibodies post-vaccination against many infectious diseases is also different where some of vaccines induce long-term antibodies while some need booster dose(s). 

 3. The practice of booster or additional dose is common for covid-19 and it would be better if the authors could see the levels of antibody decrease after the third/booster dose. If it is not possible this can be mentioned as a potential limitation of the present study.

**RESPONSE: **Please be aware that this study was conducted early of the COVID-19 pandemic where the booster dose is still questioned. Following the present study, we are continuing to measure the level of anti-SARS-CoV-2 RBD total antibody after the booster dose. We are still preparing the results of the study. We also included this as a future direction: ".... therefore further study measuring the anti-RBD antibody after the booster dose of CoronaVac or other types of COVID-19 vaccines will provide better understanding of the antibody dynamics."

 4. What are the findings about other vaccines for Covid-19? Are there any studies that have assessed antibody levels among vaccinated peoples who have been given other vaccines?

**RESPONSE: **Thank you for the question. The present study only focus on the CoronaVac and did not included those who vaccinated with other types of COVID-19 vaccines. However, we have included this  in future direction. We included: "In this study, we only determined anti-SARS-CoV-2 RBD total antibody among those who completed the primary doses of CoronaVac; therefore further study measuring the anti-RBD antibody after the booster dose of CoronaVac or other types of COVID-19 vaccines will provide better understanding of the antibody dynamics." 

 5. How can we prevent the significant loss of vaccine acquired immunity? What the authors are proposing to prevent/prolong the immunity among vaccinated individuals?

**RESPONSE: **Thank you for the question. This question is not part of the focus of the present study and authors believe more studies are needed to address the question. 

**Minor points**

 1. Please add a separate section just after discussion about the potential limitations and future direction based on the present findings considering the current weakness.

**RESPONSE: **We have included the limitation in our last paragraph of the Discussion. As per journal guideline, the limitations can be placed at the end of the Discussion. 

 2. In conclusion, we should not repeat the results, there should have some implications of the present findings in theory and practice.

**RESPONSE: **We would like to thank you for the suggestions. We have revised our conclusion more concise. The implication of this study on the practice is presented: "This may contribute to future vaccination policy management to improve and prolong the protective strategy through vaccination." Unfortunately we could not provide recommendations in the real practise since more solid data is required. 

 Best regards,

 Authors
